# Dietary protein in weight management: a review proposing protein spread and change theories

**DOI:** 10.1186/1743-7075-9-81

**Published:** 2012-09-12

**Authors:** John D Bosse, Brian M Dixon

**Affiliations:** 1 USANA Health Sciences, Inc, 3838 West Parkway Boulevard, Salt Lake City, UT, 84120, USA; 2 Division of Nutrition, University of Utah, 250 South 1850 East #214, Salt Lake City, UT, 84112, USA

**Keywords:** Protein, Habitual protein intake, Weight loss, Weight maintenance, Body composition

## Abstract

A large volume of human clinical data supports increased dietary protein for favorable changes to body composition, but not all data are conclusive. The aim of this review is to propose two theories, “protein spread theory” and “protein change theory” in an effort to explain discrepancies in the literature. Protein spread theory proposed that there must have been a sufficient spread or % difference in g/kg/day protein intake between groups during a protein intervention to see body composition and anthropometric differences. Protein change theory postulated that for the higher protein group, there must be a sufficient change from baseline g/kg/day protein intake to during study g/kg/day protein intake to see body composition and anthropometric benefits. Fifty-one studies met inclusion criteria. In studies where a higher protein intervention was deemed successful there was, on average, a 58.4% g/kg/day between group protein intake spread versus a 38.8% g/kg/day spread in studies where a higher protein diet was no more effective than control. The average change in habitual protein intake in studies showing higher protein to be more effective than control was +28.6% compared to +4.9% when additional protein was no more effective than control. Providing a sufficient deviation from habitual intake appears to be an important factor in determining the success of additional protein in weight management interventions. A modest increase in dietary protein favorably effects body composition during weight management interventions.

## Introduction

Annual healthcare costs relating to obesity approximate $150 billion in the US alone [1]. Thus, there would be great utility for dietary strategies that require minimal restriction yet benefit body composition and metabolic health. Manipulation of dietary macronutrient intake in favor of protein has shown considerable promise since the 1990s [2] and has gained increasing support recently [3-7].

In the US, the Food and Nutrition Board provides a dietary protein recommendation for adults of 0.8 g/kg/day known as the Recommended Dietary Allowance (RDA). The World Health Organization (WHO) recommends 0.83 g/kg/day of high quality protein [8]. Multiple researchers support the consumption of greater protein than the RDA, arguing that the RDA is a *minimum* level for health, not an *optimal* intake for health indicators such as body composition [9,10] something the WHO also notes [8]. Still, there is resistance to recommending a higher amount of protein to the public.

While some will critique that the satiating effect of higher dietary protein sometimes results in voluntary hypophagia [11], leading to an energy intake discrepancy between groups, there is evidence that increased dietary protein leads to improved body composition and anthropometrics under iso-, hypo-, and hyper-caloric conditions [2,11-44]. Thus, the traditional dogma of “energy in versus energy out” explaining weight and body compositional change is not entirely accurate. Another critique is that there are some studies in which greater protein is no more effective than control [45-60]. These studies do not find negative effects on body composition from higher protein, rather benefits are shown that are the same, but no greater than in controls [45-60]. There has been little examination of why discrepancies in the protein and weight management literature exist.

Due primarily to limitations of dietary adherence in free-living adults, spread, or difference, in protein intake between groups during a study is often less than originally designed [45,46,57,61]. While this would seem an intuitive explanation for why some studies do not show greater body composition and anthropometric benefits of higher protein intakes it seems to have been largely overlooked until recently [62]. One purpose of the present review is to expand upon this observation in methodological critique we have coined protein spread theory.

Additionally, the body’s response to protein is not static, but adjusts to the diet it is afforded [63-65]. For example, progressive increases in protein intake are coupled with increased fasting nitrogen losses [66,67] along with an increase in feeding induced nitrogen accrual [66,67] that is perhaps even more pronounced than fasting losses [66]. Although not fully elucidated, a possible implication of this might be an effect on lean tissue mass. A few studies specifically address change in habitual protein intake. Soenen et al. had participants increase habitual protein intake 16%, from 1.13 g/kg/day to 1.31 g/kg/day via substitution of ~500 kcal with a milk protein based supplement containing 52 g protein. Over 12 weight-stable wk this led to 0.7 kg greater lean mass gain and fat loss compared to isoenergetic controls [68]. Bray et al. reported that increasing a 1.2 g/kg/day protein intake to ≥ 1.8 g/kg/day via overfeeding led to an ~3.5-4 kg greater gain in lean body mass in eight wk [69]. Additionally, Petzke et al. reported a positive correlation (r = 0.643, p = 0.0001) between change in habitual protein intake and change in fat-free body mass [70]. While the aforementioned data point to a dynamic response to dietary protein intake it is difficult to extrapolate these findings from a healthy population to the obese. Thus, the second purpose of this review was to propose and examine protein change theory in effort to extend these findings. Objectives of protein change theory are to 1) critique the failure to assess baseline dietary intake in many studies; 2) critique what we feel in an overemphasis on % energy from protein 3) increase recognition that the response of an individual to a diet is influenced by their previous dietary exposures.

## Methods

Protein spread theory postulated that there must have been a sufficient spread or% difference in g/kg/day protein intake between groups during a protein intervention to see anthropometric differences. Protein change theory postulated that for the higher protein group, there must be a sufficient change from baseline g/kg/day protein intake to during study g/kg/day protein intake to see anthropometric benefits. Given variety of outcome measures reported in studies in this review (Table 1) categorization was necessary. “Anthropometric benefits” referred to herein are: weight loss, body-fat loss, waist circumference reduction, regional body-fat loss, lean mass preservation, decreased weight regain, decreased fat regain, or lean mass gain.

**Table 1:** Summary of 51 studies reviewed on protein and weight management in overweight and obese adults

	**Baseline**				**During study**						**Change**						
**Reference**	**BMI**	**% BF**	**Protein**	**E**	**Sex**	**Wk**	**Protein**	**Protein**	**E**	**Dsn**	**FFM**	**LM**	**% BF**	**Fat mass**	**VAT**	**BW**	**WC**
	**kg/m2**	**%**	**g/kg**	**kcal**			**g/kg**	**type**	**kcal**		**kg or %**	**kg or %**	**%**	**kg or %**	**kg or cm2**	**kg or %**	**cm**
Abete, 2009 [12]	31.4 ± 3.5	28 ± 5	NR	NR	M	8	0.84	Mix	1675	WL	-2.7 ± 1.3%	NR	NR	-12.7 ± 7.2%	NR	-5.5 ± 2.5%	-6.1 ± 2.9%
	33.2 ± 1.9	30 ± 3	NR	NR	M	8	1.4	Mix	1926	WL	-4.9 ± 1.6%	NR	NR	-18.6 ± 3.3%	NR	-8.4 ± 1.2%	-9.8 ± 2.4%
Aldrich, 2011 [13]^1,^^8^	29.9 ± 0.6	43 ± 2.5	NR	NR	F,M	20	0.95	Mix	1600	WL	NR	-0.32 ± 0.4	NR	-5.45 ± 1.1	NR	-6.1 ± 0.82	NR
	30.3 ± 0.7	42.7 ± 2.5	NR	NR	F,M	20	1.35	Mix	1605	WL	NR	0.43 ± 1.1	NR	-7.54 ± 1.4	NR	-7.6 ± 1.72	NR
	30.6 ± 0.6	45.2 ± 2.9	NR	NR	F,M	20	1.4	↑W	1600	WL	NR	-1.09 ± 0.1	NR	-8.77 ± 1.3	NR	-9.7 ± 1.27	NR
Baer, 2011 [14]^1^	31.1 ± 2.5	NR	NR	NR	M ≈ F	23	0.83	Mix	2164	WL	NR	a	NR	a	NR	a	a
	31 ± 2.2	NR	NR	NR	M ≈ F	23	1.5	↑Soy	2267	WL	NR	a	NR	ab	NR	ab	a
	30.9 ± 2.3	NR	NR	NR	M ≈ F	23	1.44	↑W	2183	WL	NR	a	NR	b	NR	b	b
Ballesteros-Pamar, 2009 [45]^4,^^7^	32.9 ± 1.9	33.6 ± 8.1	1.32	2379	F,M	16	0.86	Mix	1653	WL	-3.5%	NR	NR	-2.3%	NR	-7.3	-7.15
	32.6 ± 2.3	34.5 ± 6.5	1.24	2274	F,M	16	1.16	Mix	1797	WL	-2.9%	NR	NR	-4.7%	NR	-9	-6.4
	32.9 ± 1.9	33.6 ± 8.1	1.32	2379	F,M	16	0.86	Mix	1653	WL	-3.5%	NR	NR	-2.3%	NR	-8.7	-7.15
	32.6 ± 2.3	34.5 ± 6.5	1.24	2274	F,M	16	1.16	Mix	1797	WL	-2.9%	NR	NR	-4.7%	NR	-9.5	-6.4
Brinkworth, 2004 [46]^3,^^5^	33.6 ± 0.8	NR	NR	NR	F,M	68	1.02	Mix	1994	WLWM	NR	-0.1	NR	-2.6	NR	-2.9 ± 3.6%	NR
	34.6 ± 0.9	NR	NR	NR	F,M	68	1.22	Mix	1875	WLWM	NR	-0.4	NR	-4.2	NR	-4.1 ± 5.8%	NR
Claessens, 2009 [15]^4^	32.4 ± 1.2	39.2 ± 1.9	0.98	2398	F,M	18	0.75	Mix	1868	WM	0.96 ± 0.38	NR	-0.14 ± 0.47	0.24 ± 0.7	NR	1.19 ± 0.90	0.41 ± 0.92
	32.9 ± 1.6	42 ± 1.3	0.97	2045	F,M	18	1.68	↑C	1848	WM	0.16 ± 0.53	NR	-1.18 ±0.58	-1.55 ± 0.69	NR	-1.39 ± 0.89	-2.28 ± 0.83
	33.4 ± 1	41.2 ± 1.4	0.92	2252	F,M	18	1.65	↑W	1812	WM	1.43 ± 0.49	NR	-2.4 ± 0.67	-2.29 ± 0.75	NR	-0.85 ± 0.80	-1.73 ± 1.06
Clifton, 2008 [16]	31.8 ± 5.9	NR	NR	NR	F	52	0.85	Mix	1486	WLWM	NR	NR	NR	-2.7 ± 3.1	NR	-3.4 ± 4.4	NR
	33.1 ± 3.5	NR	NR	NR	F	52	1.24	Mix	1659	WLWM	NR	NR	NR	-4.7 ± 4.2	NR	-6.5 ± 7.5	NR
Delbridge, 2009 [47]^5^	38.6 ± 0.8	42.4 ± 1	0.88	NR	M ≈ F	52	0.81	Mix	1568	WM	0.89 ± 0.43	NR	NR	3.2 ± 1.4	NR	4.3 ± 1.4	0.92 ± 1.5
	39.3 ± 0.8	41.7 ± 1	0.89	NR	M ≈ F	52	0.95	Mix	1568	WM	0.34 ± 0.58	NR	NR	4.2 ± 2.2	NR	3 ± 1.1	-0.81 ± 1
Demling, 2000 [17]	NR	27 ± 1.8	0.76	2350	M	12	0.83	Mix	2167	WL	NR	-0.4 ± 0.4	-2	-2.5 ± 0.5	NR	-2.5 ± 0.6	NR
	NR	26 ± 1.7	0.71	2300	M	12	1.41	↑C+Ex	2167	WL	NR	-4.1 ± 1.4	-8	-7 ± 2.1	NR	-2.8 ± 0.6	NR
	NR	27 ± 1.6	0.73	2350	M	12	1.44	↑W+Ex	2183	WL	NR	-2 ± 0.7	-4	-4.2 ± 9	NR	-2.3 ± 0.5	NR
De Souza, 2012 [48]^1,^^4,^^5^	~32.78	NR	0.97	2049	F,M	104	0.79	Mix	1574	WL	a	a	a	a	a	a	NR
	~32.78	NR	0.92	1952	F,M	104	0.88	Mix	1543	WL	a	a	a	a	a	a	NR
Due, 2004 [18]^3,^^5^	30.8 ± 0.9	NR	1.01	2365	F,M	52	0.82	Mix	2221	WLWM	NR	-0.4 ± 0.8	NR	-3.1 ± 1.7	-10.5 ± 10.4 cm2	-4.3 ± 2.1	-1.8 ± 3.7
	30 ± 0.9	NR	1.05	2269	F,M	52	1.44	Mix	2173	WLWM	NR	-0.9 ± 0.9	NR	-4.6 ± 1.9	-22 ± 7 cm2	-6.2 ± 2.4	-8.4 ± 2.1
Evans, 2012 [19]^2,^^5^	NR	NR	0.88	2064	F,M	52	0.74	Mix	1590	WLWM	NR	-4.25	NR	-6.45	NR	-10.6	NR
	NR	NR	1.03	2405	F,M	52	1.26	Mix	1678	WLWM	NR	-1.85	NR	-6.35	NR	-8.75	NR
Farnsworth, 2003 [20]^2,^^3^	~34.05	NR	NR	NR	F,M	16	0.69	Mix	1756	WLWM	NR	-1.9	NR	-7.35	-3.05 kg	-8.5	NR
	~34.1	NR	NR	NR	F,M	16	1.19	Mix	1708	WLWM	NR	-1.3	NR	-7.8	-3.65 kg	-9	NR
Flechtner-Mors, 2010 [21]^5^	36.3 ± 5	NR	0.66	1627	F,M	52	0.62	Mix	1256	WL	NR	NR	NR	-3.86	NR	-6.41 ± 5.4	-8.2
	36.2 ± 4.4	NR	0.73	1705	F,M	52	0.99	Mix	1187	WL	NR	NR	NR	-7.21	NR	-8.96 ± 6.38	-12.1
Frestedt, 2008 [22]	35.4 ± 0.7	NR	0.79	1829	M ≈ F?	12	0.59	Mix	1383	WL	NR	-1.55 ± 0.39	NR	-1.62 ± 0.33	NR	-3.24 ± 0.47	-5.34 ± 0.97
	35.7 ± 0.7	NR	0.74	1893	M ≈ F?	12	0.78	↑W	1461	WL	NR	-0.75 ± 0.34	NR	-2.81 ± 0.38	NR	-3.82 ± 0.55	-6.22 ± 0.84
Gilbert, 2011 [49]	32.8 ± 2.4	48.7 ± 4.8	0.94	1867	F	26	0.79	Mix	1514	WL	-0.8	NR	-2.8	-5	NR	-5.8	-6
	33.3 ± 3.6	45.7 ± 3.7	1.08	2047	F	26	0.94	↑D	1556	WL	-1	NR	-4.1	-6	NR	-8	-7
Hinton, 2010 [50]^2,^^10^	~34.3	~44.7	0.88	1349	F,M	36	0.90	Mix	1684	WM	NR	NR	NR	NR	NR	NR	NR
	~34.3	~44.7	0.91	1314	F,M	36	1.12	↑D	2018	WM	NR	NR	NR	NR	NR	NR	NR
Hursel, 2009 [23]^9^	29.6 ± 2.1	37.3 ± 4.7	NR	NR	M ≈ F	17	0.78	Mix	Ind	WM	1	NR	1.1	2	NR	3	3
	29.5 ± 1.9	37.7 ± 3.9	NR	NR	M ≈ F	17	1.19	Mix	Ind	WM	0.8	NR	-0.6	-0.3	NR	0.5	0.2
Johnston, 2004 [51]	28.7 ± 2	NR	NR	NR	F,M	6	0.82	Mix	1700	WL	NR	NR	NR	-10.6 ± 1.4%	NR	-5.9 ± 0.5%	NR
	29.1 ± 2.6	NR	NR	NR	F,M	6	1.63	Mix	1700	WL	NR	NR	NR	-8.9 ± 2.2%	NR	-5.7 ± 0.6%	NR
Josse, 2011 [24]^1,^^4,^^6^	31.5 ± 0.6	39.1 ± 0.9	0.82	1830	F	16	0.66	Mix	1320	WL	NR	-0.7 ± 0.3	NR	a	a	NR	NR
	31.8 ± 0.6	40.6 ± 0.7	0.77	1822	F	16	0.77	↑D	1430	WL	NR	-0.2 ± 0.2	NR	a	ab	NR	NR
	31.4 ± 0.6	40.5 ± 0.6	0.8	1837	F	16	1.25	↑↑D	1500	WL	NR	0.7 ± 0.3	NR	b	b	NR	NR
Larsen, 2010 [25]^4^	NR	~35.9	1.15	2284	M ≈ F?	26	0.78	Mix	1539	WM	1.23	NR	NR	-0.54	NR	1	0.68
	NR	~35.6	1.07	2268	M ≈ F?	26	0.97	Mix	1589	WM	0.77	NR	NR	-0.63	NR	0.01	0.42
Larsen, 2011 [52]^5^	~27-40	NR	1.16	2191	F,M	52	0.79	Mix	1512	WL	NR	NR	NR	NR	NR	-2.17	-3.35
	~27-40	NR	1.20	2125	F,M	52	1.13	Mix	1566	WL	NR	NR	NR	NR	NR	-2.23	-3.54
Lasker, 2008 [26]	33.4 ± 0.7	38.2 ± 6.9	0.93	2185	F,M	16	0.71	Mix	1403	WL	NR	NR	-5.7	-4.4 ± 0.5	NR	-6.9 ± 0.8	NR
	33.8 ± 1.1	36.4 ± 7.7	0.98	2377	F,M	16	1.26	Mix	1578	WL	NR	NR	-8.7	-6 ± 0.6	NR	-9.1 ± 0.9	NR
Layman, 2003 [27]	~30.3 ± 1	NR	0.88	1959	F	10	0.79	Mix	1659	WL	NR	-1.2 ± 0.6	NR	-4.7 ± 0.7	NR	-7 ± 1.4	NR
	~30.3 ± 1	NR	0.88	1959	F	10	1.47	Mix	1670	WL	NR	-0.9 ± 0.3	NR	-5.6 ± 0.5	NR	-7.5 ± 1.4	NR
Layman, 2005 [28]^4^	35.4 ± 1.1	NR	0.93	2025	F	16	0.61	Mix	1284	WL	NR	-2.7	NR	-5	NR	-7.8	NR
	30.2 ± 1.3	NR	0.93	1905	F	16	0.71	Mix+Ex	1348	WL	NR	-1	NR	-5.5	NR	-6.7	NR
	34.8 ± 1.8	NR	1.06	2123	F	16	1.21	Mix	1448	WL	NR	-2	NR	-5.9	NR	-8.7	NR
	31.4 ± 1.7	NR	0.93	1997	F	16	1.19	Mix+Ex	1323	WL	NR	-0.4	NR	-8.8	NR	-9.8	NR
Layman, 2009 [29]^3^	32.7 ± 0.5	NR	0.89	2097	M ≈ F	52	0.74	Mix	1553	WLWM	NR	-2.7 ± 0.4	NR	-5.3 ± 0.6	NR	-8.4 ± 0.9	NR
	32.2 ± 0.5	NR	1.06	2403	M ≈ F	52	1.26	Mix	1661	WLWM	NR	-2.6 ± 0.4	NR	-7.3 ± 0.9	NR	-10.4 ± 1.2	NR
Leidy, 2007 [31]^5^	30.5 ± 0.6	44.6 ± 0.6	NR	NR	F	12	0.82	Mix	1515	WL	NR	-2.8 ± 0.5	-3.4 ± 0.5	-6.6 ± 0.6	NR	-9.5 ± 1	NR
	30.7 ± 0.9	44.2 ± 0.9	NR	NR	F	12	1.41	Mix	1550	WL	NR	-1.5 ± 0.3	-4.4 ± 0.6	-6.6 ± 0.4	NR	-8.1 ± 0.4	NR
Lejeune, 2005 [30]	27.3 ± 2.6	35.4 ± 6.9	NR	NR	M ≈ F?	26	1.07	Mix	Ind	WM	1.2	NR	0.8	1.8	NR	3	0.6
	27 ± 2.3	35.6 ± 6.7	NR	NR	M ≈ F?	26	1.33	↑C	Ind	WM	1.6	NR	-1.8	-1	NR	0.8	-1.3
Lockwood, 2008 [32]^5^	26.7 ± 1.2	29 ± 2.2	1	2039	M ≈ F	10	0.91	Mix+Ex	1986	WL	0.8 ± 0.6	NR	-1.2 ± 0.4	-1.1 ± 0.4	NR	-0.3 ± 0.5	NR
	29.2 ± 1.5	34.1 ± 1.3	1.02	2166	M ≈ F	10	1.38	↑W&C+Ex	1860	WL	0.9 ± 0.5	NR	-2.5 ± 0.4	-2.7 ± 0.4	NR	-1.8 ± 1	NR
Luscombe, 2002 [53]^3^	32.6 ± 1.4	37.8 ± 2	NR	NR	F,M	12	0.74	Mix	1680	WLWM	NR	NR	a	a	a	NR	-4.3 ± 0.7
	33.9 ± 1.2	42.2 ± 2.2	NR	NR	F,M	12	1.27	Mix	1715	WLWM	NR	NR	a	a	a	NR	-4.9 ± 0.4
Luscombe, 2003 [54]^3^	33.5 ± 0.9	NR	NR	NR	F,M	16	0.73	Mix	1779	WLWM	a	a	a	a	a	-8 ± 0.7	a
	34.8 ± 1	NR	NR	NR	F,M	16	1.24	Mix	1723	WLWM	a	a	a	a	a	-7.9 ± 1.1	a
Magrans-Courtney, 2011 [55]^5^	NR	46.3 ± 4	0.92	1987	F	14	0.89	Mix	1832	WL	0.5	NR	-1.8	-2.1	NR	-1.9	NR
	NR	45.9 ± 2	0.81	1746	F	14	1.07	Mix	1537	WL	-0.2	NR	-1.5	-2.4	NR	-2.5	NR
Mahon, 2007 [33]	28.4 ± 3.3	43.7 ± 5.1	0.99	1699	F	9	0.63	Mix	1158	WL	-1.7 ± 1	NR	-2.1 ± 1.5	-3.9 ± 1.5	NR	-5.6 ± 1.8	NR
	29.1 ± 4.3	42.9 ± 4.1	0.89	1579	F	9	0.88	↑Ch	1098	WL	-2.3 ± 1	NR	-3.3 ± 1.7	-5.6 ± 2.2	NR	-7.9 ± 2.6	NR
	30.1 ± 3.1	43.4 ± 5.1	0.99	1862	F	9	0.88	↑B	1114	WL	-2.2 ± 1.3	NR	-2.1 ± 1.8	-4.3 ± 2.1	NR	-6.6 ± 2.7	NR
McAuley, 2005 [34]^3^	36.6 ± 5.6	NR	0.83	1812	F	24	0.78	HiCarb	1433	WLWM	-2.1	NR	NR	-3.9	NR	-4.7	-6.9
	36 ± 3.9	NR	0.85	1874	F	24	1.05	HiFat	1450	WLWM	-5.2	NR	NR	-5.2	NR	-7.1	-9.8
	34.5 ± 5.3	NR	0.94	2006	F	24	1.04	HiPro	1577	WLWM	-2.8	NR	NR	-4.4	NR	-6.9	-8.8
McMillan-Price, 2006 [35]	30.9 ± 0.6	NR	1.15	2300	F,M	12	0.73	HiGI	1435	WL	NR	-0.5 ± 0.2	NR	-2.8 ± 0.5	NR	-3.7 ± 0.5	-4.3 ± 0.7
	31.3 ± 0.8	NR	1.01	2202	F,M	12	1.08	HiGI	1421	WL	NR	-0.6 ± 0.2	NR	-4.3 ± 0.5	NR	-5.3 ± 0.5	-6.3 ± 0.6
Meckling, 2007 [36]^4^	28.7 ± 2.3	38.4 ± 6.4	0.71	1773	F	12	0.71	Mix	1391	WL	NR	0.8	-2.5	-3.7	NR	-2.1	NR
	29.2 ± 3.5	39.5 ± 5.9	0.71	1773	F	12	0.73	Mix+Ex	1260	WL	NR	1.2	-4.3	-4.1	NR	-4	NR
	31.2 ± 3.5	42.4 ± 4.6	0.71	1773	F	12	1	Mix	1383	WL	NR	0.9	-4.6	-5.2	NR	-4.6	NR
	30.8 ± 4.7	40.8 ± 5.8	0.71	1773	F	12	1.33	Mix+Ex	1217	WL	NR	0.5	-5.7	-7.4	NR	-7	NR
Mojtahedi, 2011 [11]	32.7 ± 4.2	NR	0.93	1743	F	26	0.81	Mix	1627	WL	NR	0.7%	NR	NR	NR	-3.1	NR
	32.3 ± 3.9	NR	0.89	1687	F	26	1.07	↑W	1369	WL	NR	2.3%	NR	NR	NR	-6.7	NR
Morenga, 2010 [43]^5^	32.5 ± 5.3	46.1 ± 6.1	1.02	2068	F	10	0.97	Mix	1752	WL	NR	-0.2	-0.1	-0.1	NR	-0.2	-0.8
	32.3 ± 5.6	45.6 ± 6	0.99	1990	F	10	1.24	HiFib	1756	WL	NR	-0.1	-0.6	-1	NR	-1.5	-2.2
Navas-Carretero, 2011 [38]^1^	28.6 ± 4.3	29.5 ± 8.1	NR	NR	M ≈ F?	4	0.95	Mix	1710	WL	NR	NR	NR	a	NR	a	NR
	28.6 ± 4.3	29.5 ± 8.1	0.95	1710	M ≈ F?	4	1.21	Mix+Sn	1815	WL	NR	NR	NR	b	NR	b	NR
Nickols-Richardson, 2005 [39]	31.1 ± 4.9	NR	1.12	2340	F	6	0.79	Mix	1395	WL	NR	NR	NR	NR	NR	-4.2	NR
	30.3 ± 5.5	NR	0.89	2025	F	6	1.11	Mix	1420	WL	NR	NR	NR	NR	NR	-6.4	NR
Noakes, 2005 [40]	33 ± 4	NR	NR	NR	F	12	0.65	Mix	1247	WL	NR	-1.8 ± 0.3	NR	-4.5 ± 0.5	NR	-6.9 ± 0.5	NR
	32 ± 6	NR	NR	NR	F	12	1.14	Mix	1268	WL	NR	-1.5 ± 0.3	NR	-5.7 ± 0.6	NR	-7.6 ± 0.4	NR
Papakonstantinou, 2010 [41]	34 ± 1	NR	1.06	2041	F,M	4	0.66	Mix	1550	WL	-1	NR	NR	-2	NR	-3	-4
	33 ± 1	NR	1.08	2041	F,M	4	1.27	Mix	1545	WL	-0	NR	NR	-3	NR	-3	-2
Parker, 2002 [42]^2^^3^	~33.3	NR	NR	NR	F,M	12	0.78	Mix	1664	WLWM	NR	-1.35	NR	-3.65	NR	-4.8	NR
	~35	NR	NR	NR	F,M	12	1.35	Mix	1808	WLWM	NR	-0.52	NR	-4.25	NR	-5.5	NR
Rizkalla, 2012 [56]	~31.86	NR	0.89	1878	M,F	4	0.77	Mix	1283	WL	-1.74	NR	NR	-1.1	-0.13 kg	-2.74	-0.13
	~31.86	NR	0.84	1630	M,F	4	1.1	Mix	1199	WL	-2.09	NR	NR	-1.7	-0.81 kg	-3.56	-0.81
Sacks, 2009 [57]^1^^5^	33 ± 4	NR	0.97	2014	F,M	104	0.79	Mix	1574	WL	NR	NR	NR	NR	NR	-3.6	a
	33 ± 4	NR	0.93	1921	F,M	104	0.9	Mix	1542	WL	NR	NR	NR	NR	NR	-4.5	a
Sargrad, 2005 [58]	36 ± 3	39.5 ± 2.5	NR	NR	F,M	8	0.70	Mix	1371	WL	+0.1	NR	NR	-2.2 ± 0.7	NR	NR	-2.2 ± 0.9
	33 ± 2	42.4 ± 3.1	NR	NR	F,M	8	0.92	Mix	1274	WL	+0.4	NR	NR	-2.6 ± 1.8	NR	NR	-2.5 ± 1.6
Skov, 1999 [2]^5^	30.8 ± 0.4	NR	NR	NR	F,M	26	0.89	Mix	2603	WL	NR	NR	NR	-4.3 ± 1.2	-16.8 cm2	-5 ± 1.4	NR
	30 ± 0.4	NR	NR	NR	F,M	26	1.5	Mix	2138	WL	NR	NR	NR	-7.6 ± 1.4	-33 cm2	-8.7 ± 1.4	NR
Sukumar, 2011 [59]	NR	NR	0.85	1672	F	52	0.73	Mix	1375	WL	NR	-1.4	NR	-4.5	NR	-6.1	NR
	NR	NR	0.9	1733	F	52	0.98	Mix	1480	WL	NR	-1.2	NR	-4.2	NR	-5.7	NR
Te Morenga, 2011 [43]	34.2 ± 4.8	NR	0.93	2027	F	8	0.82	HiFib	1427	WL	NR	-0.4 ± 0.5	-1.5 ± 0.8	-2.5 ± 1	NR	-3.3 ± 0.9	-4.7 ± 1.1
	33.7 ± 4.9	NR	0.9	1940	F	8	1.14	Mix	1555	WL	NR	-0.2 ± 0.4	-2.7 ± 0.5	-4 ± 0.6	NR	-4.5 ± 0.8	-5.4 ± 0.9
Westerterp-Plantenga, 2004 [44]	27.6 ± 2.6	35.3 ± 6.7	NR	NR	M ≈ F?	13	1.29	Mix	2699	WM	0.8	NR	0.6	1.2	NR	2	1
	27 ± 2.4	35.4 ± 6.4	NR	NR	M ≈ F?	13	1.71	↑C	2962	WM	2	NR	-1.8	-1	NR	1	1
Wycherley, 2010 [60]^4^	34.8 ± 4.9	NR	NR	NR	M ≈ F?	14	0.71	Mix	1499	WL	-2.2 ± 1.9	NR	NR	-6.5 ± 3.7	NR	-8.6 ± 4.6	-8.2 ± 4.6
	34.9 ± 4.9	NR	NR	NR	M ≈ F?	14	0.65	Mix+Ex	1481	WL	-2.4 ± 2.5	NR	NR	-8.1 ± 3.8	NR	-10.5 ± 5.1	-11.3 ± 4.6
	35.6 ± 3.8	NR	NR	NR	M ≈ F?	14	1.16	Mix	1510	WL	-1.9 ± 1.5	NR	NR	-7.1 ± 4	NR	-9 ± 4.8	-8.9 ± 3.9
	36.6 ± 5	NR	NR	NR	M ≈ F?	14	1.09	Mix+Ex	1514	WL	-2.4 ± 3.1	NR	NR	-11.4 ± 3.9	NR	-13.8 ± 6	-13.7 ± 4.6

1 Changes without a common letter differ. Provided when absolute values could not be accurately determined from information given.

2 Some data provided were divided by gender and were averaged.

3 Intake data divided by WL and WM phases and were averaged.

4 Multiple LP and HP groups; data for each protein level were averaged since significant differences were observed or not observed between all LP and HP levels.

5 intake data and/or outcome measure data reported for multiple time points were averaged.

6 ↑↑ denotes an even greater level of intake than another group with an increased intake of the same protein type.

7 Denotes weight loss data reported differently in two tables; both data sets are reported.

8 Regional leg and gynoid fat losses significantly greater in ↑ W group vs. control.

9 Urinary marker derived protein intake, not dietary recall data provided.

10 Results provided in per gender fashion such that it was clear there were no differences and this was stated, however, specific anthropometric change numbers could not be derived from the data provided.

~, approximate BMI or BF% (original data reported as one baseline mean for all participants); B, beef; C, casein; Ch, chicken; D, dairy; Dsn, study design; Dur, duration; E, energy; Ex, exercise; F, M, more females in group than males; HiFib, high fiber; Ind, caloric allotment calculated individually based on baseline characteristics of each participant; M ≈ F, gender distribution nearly equal; M ≈ F?, n of each gender not reported in mixed gender studies; Mix, mixed diet with varied protein sources; NR, not reported; Sn, additional protein containing snacks; W, whey; Wk, weeks; WL, weight loss only study; WM, weight maintenance (of previous loss) – protein intervention only applied to WM period – outcome data only reported for WM period; WLWM, study comparing protein intervention spanning both weight loss and weight maintenance periods, data reported are for whole duration.

**Figure 1:**
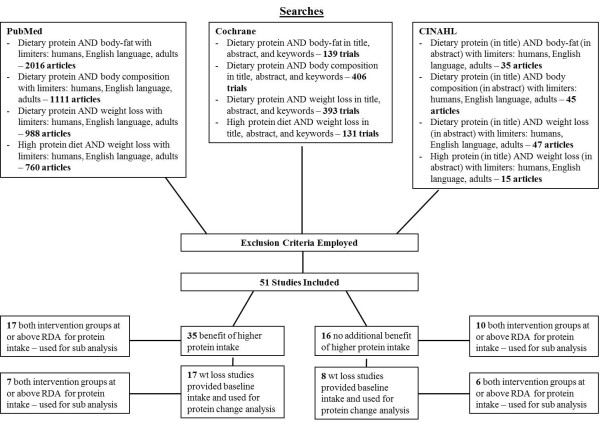
**Literature review searches used in developing “protein spread” and “protein change” theories and RDA sub-analysis.** 1 Reason for exclusion listed only once – some studies may have been excluded for meeting multiple exclusion criteria.

Keyword searches in the PubMed, Cochrane Central Register of Controlled Trials, and CINAHL databases were conducted up to July 2012 using the search criteria in Figure 1. The protein spread theory portion (Table 2) of this review examined weight loss trials with a protein intervention, weight loss trials followed by a weight maintenance period incorporating a protein intervention, and protein interventions that spanned both weight loss and weight maintenance periods. Only weight loss studies were examined in the protein change analysis (Tables 3 &4). Including weight maintenance studies would introduce a brief period where participants’ metabolisms had to adjust to an atypical intake, making “habitual protein intake” leading into the protein intervention difficult to define. Only two cross-over studies [38,56] were designed such that the habitual intake of participants prior to intervention could be determined and thus could be included in the change analysis. See the legend of Table 1 for more on study categorization.

**Table 2:** Studies suiting RDA inclusion criteria and included in protein spread theory analysis

**Benefit**		**No > benefit than control**	
**Study**	**% Spread (g/kg/day)**	**Study**	**% Spread (g/kg/day)**
**Abete, 2009**[12]	**66.7**	**Ballesteros-Pomar, 2009**[45]	**34.9**
**Aldrich, 2011**[13]	**47.4**	***Brinkworth, 2004***[46]	***19.6***
***Baer, 2011***[14]	***73.5***	***Delbridge, 2009***[47]	***17.3***
*Claessens, 2009*[15]	*121.3*	***De Souza, 2012***[48]	***11.4***
***Clifton, 2008***[16]	***45.9***	**Gilbert, 2011**[49]	**19**
**Demling, 2000**[17]	**72.3**	**Hinton, 2010**[50]	**24.4**
***Due, 2004***[18]	***75.6***	***Johnston, 2004***[51]	***98.8***
Evans, 2012 [19]	70.3	***Larsen, 2011***[52]	***43***
*Farnsworth, 2003*[20]	*72.5*	*Luscombe, 2002*[53]	*71.6*
*Flechtner-Mors*[21]	*59.7*	*Luscombe, 2003*[54]	*69.9*
*Frestedt, 2008*[22]	*32.2*	***Magrans-Courtney, 2011***[55]	***20.2***
*Hursel, 2009*[23]	*52.6*	Rizkalla, 2012 [56]	42.9
Josse, 2011 [24]	73.6	***Sacks, 2009***[57]	***13.9***
*Larsen, 2010*[25]	*24.4*	*Sargrad, 2005*[58]	*31.4*
Lasker, 2008 [26]	77.5	*Sukumar, 2011*[59]	*34.2*
***Layman, 2003***[27]	***86.1***	*Wycherley, 2010*[60]	*67.7*
Layman, 2005 [28]	81.8		
*Layman, 2009*[29]	*70.3*		
***Lejeune, 2005***[30]	***24.3***		
***Leidy, 2007***[31]	***72***		
**Lockwood, 2008**[32]	**51.6**		
*Mahon, 2007*[33]	*39.7*		
McAuley, 2005 [34]	33.3		
McMillan-Price [35]	47.9		
*Meckling, 2007*[36]	*62.5*		
**Mojtahedi, 2011**[11]	**32.1**		
**Morenga, 2010**[37]	**27.8**		
**Navas-Carretero, 2011**[38]	**27.4**		
**Nickols-Richardson, 2005**[39]	**40.5**		
*Noakes, 2005*[40]	*75.4*		
Papakonstantinou [41], 2010	92.4		
*Parker, 2002*[42]	*73.1*		
***Skov, 1999***[2]	***68.5***		
**Te Morenga, 2011**[43]	**39**		
***Westerterp-Plantenga, 2004***[44]	***32.6***		
Average% Spread (g/kg/day):	58.4	Average% Spread (g/kg/day):	38.8
**Average% Spread (g/kg/day): RDA only**	**52**	**Average% Spread (g/kg/day): RDA only**	**30.3**
*Average% Spread (g/kg/day): Urinary Biomarker only*	*62.7*	*Average% Spread (g/kg/day): Urinary Biomarker only*	*41.6*

Bold = studies meeting RDA inclusion criteria; *Italics = studies with urinary biomarker verification of protein intakes.*

Benefit = higher protein group in these studies experienced greater anthropometric benefits than did control group during the intervention; No > benefit than control = higher protein group in these studies experienced anthropometric benefits equivalent to the control group during the intervention.

**Table 3:** Protein change theory studies showing anthropometric benefits of increased protein versus control

**Study**	**LP base intake (g/kg/day)**	**LP study intake (g/kg/day)**	**HP base intake (g/kg/day)**	**HP study intake (g/kg/day)**	**LP Change (%)**	**HP Change (%)**
**Demling, 2000**[17]	**0.76**	**0.83**	**0.72**	**1.43**	**9.5**	**98.1**
Evans, 2012 [19]	0.88	0.74	1.03	1.26	−15.9	22.3
*Flechtner-Mors*[21]	*0.66*	*0.62*	*0.73*	*0.99*	*−6.1*	*35.6*
*Frestedt, 2008*[22]	*0.76*	*0.59*	*0.74*	*0.78*	*−22.4*	*5.4*
**Josse, 2011**[24]	**0.78**	**0.84**	**0.8**	**1.33**	**7.7**	**66.3**
Lasker, 2008 [26]	0.93	0.71	0.98	1.26	−23.7	28.6
Layman, 2005 [28]	0.93	0.66	0.99	1.2	−29.03	21.2
**Lockwood, 2008**[32]	**1**	**0.91**	**1.02**	**1.38**	**−9**	**35.3**
*Mahon, 2007*[33]	*0.99*	*0.63*	*0.94*	*0.88*	*−36.4*	*−6.4*
McMillan-Price [35]	1.15	0.73	1.01	1.08	−36.5	6.9
*Meckling, 2007*[36]	*0.89*	*0.72*	*0.83*	*1.17*	*−19.1*	*41*
**Morenga, 2010**[37]	**1.02**	**0.97**	**0.99**	**1.24**	**−4.9**	**25.3**
**Mojtahedi, 2011**[11]	**0.98**	**0.87**	**0.91**	**1.21**	**−11.2**	**33**
**Navas-Carretero, 2011**[38]^**1**^	**X-Over**	**X-Over**	**0.95**	**1.21**	**X-Over**	**27.4**
**Nickols-Richardson, 2005**[39]	**1.12**	**0.79**	**0.89**	**1.11**	**−29.5**	**24.7**
Papakonstantinou [41], 2010	1.06	0.66	1.08	1.27	−37.7	17.6
**Te Morenga, 2011**[43]	**0.93**	**0.82**	**0.9**	**1.14**	**−11.8**	**26.7**
Average					−16.6	28.6
**Average: RDA only**					**−9.8**	**36.9**
*Average: Urinary Biomarker only*					*−21*	*18.9*

**Bold = studies meeting RDA inclusion criteria**; *Italics = studies with urinary biomarker verification of protein intakes.*

1 X-Over – crossover design whereby the same participants increased their protein intake from a previous controlled intake period.

Only weight loss studies reporting baseline protein intake. The higher protein groups in all of these studies experienced greater anthropometric benefits than the respective control groups during the intervention.

**Table 4:** Protein change theory studies showing no > anthropometric benefits of increased protein versus control

**Study**	**LP Base Intake (g/kg/day)**	**LP Study Intake (g/kg/day)**	**HP Base Intake (g/kg/day)**	**HP Study Intake (g/kg/day)**	**LP Change (%)**	**HP Change (%)**
**Ballesteros-Pamar, 2009**[45]	**1.32**	**0.86**	**1.24**	**1.16**	**−34.8**	**−6.5**
***De Souza, 2012***[48]	***0.97***	***0.79***	***0.92***	***0.88***	***−18.6***	***−4.3***
**Gilbert, 2011**[49]	**0.94**	**0.79**	**1.08**	**0.94**	**−16**	**−13**
***Larsen, 2011***[52]	***1.16***	***0.79***	***1.2***	***1.13***	***−31.9***	***−5.8***
***Magrans-Courtney, 2011***[55]	***0.92***	***0.89***	***0.81***	***1.07***	***−3.3***	***32.1***^***1***^
Rizkalla, 2012 [56]	0.89	0.77	0.84	1.1	−13.5	31
***Sacks, 2009***[57]	***0.97***	***0.79***	***0.93***	***0.9***	***−18.6***	***−3.2***
*Sukumar, 2011*[59]	*0.85*	*0.73*	*0.9*	*0.98*	*−14.1*	*8.9*
Average					−17.6	4.9
**Average: RDA only**					**−18.9**	**−0.1**
*Average: Urinary Biomarker only*					*−17.3*	*5.5*

Bold = studies meeting RDA inclusion criteria; *Italics = studies with urinary biomarker verification of protein intakes.*

1 See discussion for explanation of the limitations of this data set.

Only weight loss studies reporting baseline protein intake. The higher protein groups in all of these studies experienced no greater anthropometric benefits than the respective control groups during the intervention.

The following were reasons for exclusion from this review: 1) examination of total protein intake not part of design (focus was on another macronutrient or timing/type of protein was manipulated in a manner not intended to effect total protein intake); 2) energy deficit not incorporated or not incorporated in both groups; 3) non-overweight/obese population; 4) significant differences in baseline anthropometrics; 4) poor dietary control or reporting; 5) < 4 wk; 6) exercise or lifestyle intervention employed not consistent between groups; 7) duplicate of another included study reporting different data sets; 8) participants with conditions not necessarily related to obesity (gout, heart failure, polycystic ovarian syndrome, AIDS, post-pregnancy or bariatric surgery, etc.). This review focused on data from the past two decades (1992-present). A recent meta-regression encompassing 1936–2005 concluded that a greater intake of dietary protein enhances maintenance of lean mass by ~0.6 to 1.2 kg during weight loss. See the analysis by Krieger et al. [3] for further reading.

Based upon the aforementioned criteria, 51 studies were reviewed (Table 1). Protein intake is related to body composition and metabolic health, and the RDA is a minimum needed for health in these areas. Thus, the inadequate protein consumed by participants (as defined by the RDA) in the lower protein group of some studies may be viewed by some scientists as creating easier circumstances for a higher protein group to see improved anthropometrics vs. this sub-optimal protein group. For this reason, study groups in which intake of the lower protein group was at or above 0.79 g/kg/day were isolated in a subsequent reanalysis. Given rounding in the calculation methods that follow, studies with a lower protein group at 0.79 g/kg/day were included as meeting the RDA.

Although not perfect, dietary recalls can be reliable in classifying macronutrient intakes [71]. Data from dietary recalls and weighed food records were used for consistency, as this was the form of protein intake reporting used in all studies. Studies using only food frequency questionnaires (FFQs) were excluded. Only some studies provided urine marker derived protein intakes. Some studies provided protein intake data in g/kg/day terms. When only% energy from protein was provided, calculations using energy intake were made to convert this value into g/kg/day. Evidence was examined in a g/kg/day fashion for a more stable comparison across variations of body mass and intakes between studies.

When only g protein/day was provided, baseline body mass was the devisor, yielding g/kg/day. Some studies providing protein intake in g/kg/day terms calculated using baseline body mass while others used post-weight loss body mass. For these studies, the authors manually derived g/kg/day protein intakes using baseline body mass for consistency. Energy intakes provided in mega joules or kilojoules were converted to kilocalories. Dietary intake data sets for multiple time points were often combined as a composite and are noted (Table 1).

The term “higher protein” was used to describe the group that had a “higher” protein intake relative to a “lower” protein group, sometimes referred to as a “control” group. “Higher” and “lower” were relative, not denoting a specific intake.

“Spread” calculations for protein spread theory were calculated by:

Between group% spread in protein intake = [((higher protein group g/kg/day intake during study - control group g/kg/day intake during study)/control group g/kg/day intake during study) × 100]

“Change in habitual protein intake” calculations were calculated by:

Change in habitual protein intake = [((g/kg/day intake during study – g/kg/day intake at baseline)/g/kg/day intake at baseline) × 100]

For both theories, after values were obtained for each study, means of particular groups of studies (Figure 1) were calculated. Baseline intake refers to g/kg/day protein intake prior to protein intervention.

## Results

Thirty-five of the 51 studies examined showed superior body composition and anthropometric benefits of a higher protein intake over control. However, sixteen studies showed no greater body composition and anthropometric benefits of a higher protein intake compared to control. We proposed protein spread theory and protein change theory as possible explanations for this discrepancy.

### Protein spread theory

Within 35 studies showing anthropometric benefits of higher protein, g/kg/day intake was 58.4% greater than control on average (Table 2). Within 16 studies showing no additional anthropometric benefits of higher protein, g/kg/day intake was only 38.8% greater than control on average.

**Figure 2:**
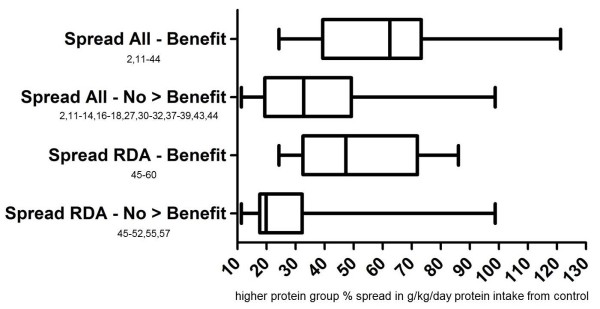
**Spreads in protein consumption between higher and lower protein groups in protein spread analysis.** Spread RDA – Benefit = only those studies meeting RDA inclusion criteria in which the higher protein group experienced greater anthropometric benefits than controls during the intervention; Spread All – Benefit = all studies in which the higher protein group experienced greater anthropometric benefits than controls during the intervention; Spread RDA –No > Benefit = only those studies meeting RDA inclusion criteria in which the higher protein group experienced no greater anthropometric benefits than controls during the intervention; Spread All – No > Benefit = all studies in which the higher protein group experienced greater anthropometric benefits than controls during the intervention.

Since some scientists may find excluding studies with a sub-RDA lower protein group a more balanced analysis of protein spread theory, a reanalysis was performed including only the 27 studies that met RDA inclusion criteria. The 27 were divided into: 1) those 17 showing additional benefit to increased protein and 2) those 10 that did not (Figure 2). This additional analysis supported protein spread theory as the mean spread in g/kg/day protein intake in the 17 studies showing a benefit of increased protein was 52%. This was close to the 58.4% figure from the original analysis (Table 2). Similarly, the mean spread in the 10 studies showing no additional benefit of increased protein was 30.3%. This was close to the 38.8% figure from the original analysis and supported protein spread theory. Benefit versus no greater benefit group means were also provided for only those studies providing urinary biomarker verification of protein intakes (Table 2).

### Protein change theory

Not all weight loss only studies reported baseline dietary intake. In those 25 that did, the average percent increase in habitual g/kg/day protein intake was 28.6% in 17 studies which showed anthropometric benefit to a higher protein intake compared to only 4.9% in eight studies that showed no additional benefit (Tables 3 &4).

**Figure 3:**
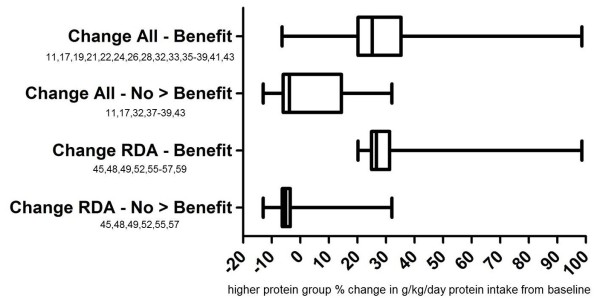
**Percent deviation from habitual protein intake among groups in protein change analysis.** Only weight loss studies reporting baseline protein intake. Change RDA – Benefit = only those studies meeting RDA inclusion criteria in which the higher protein group experienced greater anthropometric benefits than controls during the intervention; Change All – Benefit = all studies in which the higher protein group experienced greater anthropometric benefits than controls during the intervention; Change RDA –No > Benefit = only those studies meeting RDA inclusion criteria in which the higher protein group experienced no greater anthropometric benefits than controls during the intervention; Change – No > Benefit = all studies in which the higher protein group experienced greater anthropometric benefits than controls during the intervention.

Since perhaps some scientists would find excluding studies with a sub-RDA lower protein group a more balanced analysis of protein change theory, a reanalysis was performed including only the 13 baseline intake reporting studies that met RDA inclusion criteria. The 13 were divided into: 1) those seven showing additional benefit to increased protein and 2) those six that did not (Figure 3). This additional analysis supported protein change theory as the mean spread in g/kg/day protein intake in the seven studies showing a benefit of increased protein was 36.9%. This was relatively close to the 28.6% figure from the original analysis (Table 2). Similarly, the mean spread in the six studies showing no benefit of increased protein was −0.1%. This was close to the 4.9% figure from the original analysis and supported protein change theory. Benefit versus no greater benefit group means were also provided for only those studies providing urinary biomarker verification of protein intakes (Tables 3 &4).

## Discussion

This review supports our protein spread and change theories as possible explanations for discrepancies in the protein and weight management literature. Among studies showing greater anthropometric benefits of higher protein there is typically a relatively large% difference spread of approximately 58.4% between the g/kg/day intake of the higher protein group and control. Additionally, that the higher protein group’s during study g/kg/day protein intake is substantially different, or approximately 28.6% greater than baseline, is important. When these spreads and habitual deviations are lower, closer to 38.8% and 4.9% respectively, there is little additional anthropometric benefit produced by higher protein interventions. Evidence weighs heavily toward studies showing anthropometric benefits of increased protein intake [2,11-44]. Those that did not support additional benefits still showed that higher protein was equally as good as an alternative diet [45-60].

### Protein spread theory

Studies showing anthropometric benefits in the protein spread analysis had a higher protein group consuming on average 58.4% g/kg/day more protein than controls. For example, Leidy et al. had overweight and obese women maintain a caloric deficit of ~750 kcal/day for 12 wk. Controls consumed 0.82 g/kg protein/day. Higher protein participants consumed 1.41 g/kg/day. The higher protein group retained significantly more lean mass (+1.3 kg) than control, and achieved the same weight loss [31]. In another study, participants consuming 1.71 g/kg/day protein gained back 1 kg less weight, lost 2.2 kg more fat, and gained 1.2 kg more fat-free mass versus controls consuming 1.29 g/kg/day protein during 13 wk weight maintenance [44]. Similarly, during 26–52 wk weight maintenance, 3.1-3.6 and 2–3.3 kg greater weight and fat loss were achieved versus control when higher protein groups achieved protein intake spreads from controls of 45.9-68.5% [2,16].

There appeared to be some outliers within studies showing no additional benefit of a higher protein intake (Table 2), however, there appeared to be plausible explanations for nearly all outliers. Wycherley et al. [60] was grouped in the “no benefit” studies, despite showing a 2 kg greater reduction in fat mass in higher protein participants achieving a 67.7% g/kg/day spread because this fat reduction just missed statistical significance (p = 0.06). There were also similar trends for body mass and waist circumference [60]. A six wk study by Johnston et al. did not show a superior anthropometric effect of a 98.8% g/kg/day spread [51], but did not assess baseline intake and used a bioelectrical impedance device to assess body composition, shown to be problematic in short weight loss [72]. Higher protein participants did have greater diet satisfaction and less hunger [72] which influences long-term dietary success [25,29]. Although there were no greater anthropometric benefits of a 71.6% g/kg/day spread in a 12 wk study by Luscombe et al., the lower protein group contained double the # of women in the higher protein group. Meanwhile the higher protein group has more than double the urinary albumin level of lower protein participants at baseline, seeming to indicate some discrepancy between groups in protein metabolism [53]. Although there did not appear a plausible explanation why a 69.9% g/kg/day intake spread did not yield greater anthropometric benefits in a another study by Luscombe and colleagues [54] as in the previous outliers [51,53] no baseline dietary information was provided and thus it is unknown if these large between group spreads actually involved any appreciable change in habitual protein intake for the higher protein groups.

A flaw in some long duration trials was that while no differences in weight loss were shown with higher protein, body composition was not assessed. Additionally, protein intake spread between groups was often less than designed [45,46,48,57,61], a problem noted in a recent editorial [62].

### Protein change theory

Multiple studies in this review (Table 3) showed 0.8-3.3 g/kg/day greater fat loss in higher protein participants over 4–26 wk when change from habitual intake was 20.2%-35.3% [11,32,38,43]. There appeared to be three outliers in Table 3[22,33,35]. Higher protein participants in these studies achieved changes in habitual protein intake of only 5.4, -6.4, and 6.9% respectively yet still saw greater anthropometric improvements compared to controls. However, these studies involved appreciable g/kg/day protein intake spreads of 32.2, 39.7, and 47.9% respectively. Perhaps this spread, coupled with the fact that the lower protein groups in Mahon et al. and McMillan Price et al. reduced their habitual protein intakes the most of any studies in this review, -36.4 and −36.5% respectively, was a combination that allowed for superior anthropometric outcomes for these higher protein participants. Although not as pronounced, lower protein participants in the Frestedt et al. study notably decreased their habitual protein intake by −22.4%, leading to the lowest during study lower protein group intake in this review of 0.59 g/kg. Perhaps this coupled with the aforementioned spread was enough to allow for anthropometric differences between protein groups. Additionally in regard to the McMillan-Price et al. study [35], participants were stratified: 1) lower protein/higher GI; 2) lower protein/lower GI; 3) higher protein/higher GI; and 4) higher protein/lower GI [35]. In women, higher protein/higher GI lost significantly more body and fat mass than lower protein/higher GI. There was a 47.9% g/kg/day protein intake spread between these groups. There was also a small 6.74% increase in habitual protein intake for the higher protein/higher GI group. Conversely, higher protein/lower GI was less effective for weight and fat loss compared to lower protein/lower GI. Results were puzzling as lower GI can aid weight management. However, spread in protein intake between low GI groups was only 32.8% and higher protein/lower GI did not change their habitual intake (± 0%). Thus, three of the four theory related means nearly fit our mean theory numbers, with all four fitting directionally. Some have shown gender difference in response to higher protein [20,42] while others have not [23,44].

In table 4 there appeared to be two outliers within studies showing no additional benefit of a higher protein intake, however, there appeared to be plausible explanations for both. Higher protein participants in a study by Rizkalla et al. increased their habitual protein intake by 31% and achieved a greater reduction in waist circumference (p = 0.07), trunk fat (p = 0.08), total fat (p = 0.10), body-weight (p = 0.14), and adipocyte diameter (p = 0.048). This study [56] was grouped in the “no benefit” studies because only the adipocyte diameter finding was statistically significant and per the methods of this review, only whole/regional body anthropometric measures could be considered “anthropometric benefits.” The higher protein group in a study by Magrans-Courtney et al. showed no greater benefit of a 32% increase in habitual protein intake. However, the increase in habitual protein intake in this higher protein group of 32% was a composite of a 55% increase at wk 10 and a 10% increase at wk 14 [55]. The reported protein intake at wk 10 had a standard deviation of ± 47 g as compared to ± 10–13 g at wk 0 and 14. Thus, the increase in habitual intake was likely closer to 10%, more in line with the 4.9% average from this group of studies (Table 4).

A flaw in previous trials was that at times higher protein groups consumed more protein than control, yet less than their habitual intake, and saw no difference in anthropometrics [33,52,57,61]. Thus, the “intervention” diet was really not an intervention to their metabolism. The human body does not know persay the% kcals it is receiving from each macronutrient. In some cases, increasing the% of kcals from protein during energy restriction can actually result in less protein being consumed during intervention than habitual intake as a simple function of energy deficit. Habitual intake mediates the effects of protein on bone health and satiety [73,74] and studies have shown that that the thermic effect of protein decreases over time while dieting [53,54]. We propose that changes in habitual protein intake may mediate the effects of protein on lean body mass [70]. Perhaps a progressive loss of body and lean body mass with dieting increases the capacity for amino acid deposition. Meanwhile this more rapid disposal of amino acids from circulation may mandate a progressive increase in protein intake to achieve satiety [74] and ultimately weight management goals.

The lack of accounting for protein distribution throughout the day may also explain outliers in this review. Two leading protein metabolism research groups have recently discussed the importance of spacing protein evenly throughout the day to optimize body composition endpoints [75,76]. Thus, it is unlikely that adding additional protein to meals that were already protein rich has the same effect as achieving a higher daily protein intake by adding protein to meals that were previously protein poor.

### New approaches in data reporting and assessment

Recently, Layman et al. and Flechtner-Mors et al. reported body composition changes as a ratio of fat lost/lean mass lost [21,27]. Westerterp-Plantenga et al. generated an energy efficiency ratio of body mass regain/energy intake [44], while Ballesteros-Pamar et al. examined the ratio of weight loss achieved/energy deficit [45]. Layman et al. and Flechtner-Mors et al. analyzed participants achieving at least 10% weight loss and found a greater prevalence of higher protein participants [21,29], while Frestedt et al. split participants into “responders” and “non-responders” [22]. If all studies reported these additional data sets and baseline dietary intakes, further insight could be gained. Although most studies in this review verified protein intakes with urinary biomarkers (Tables 2, 3, 4), the lack of these assessments in all studies is a limitation. These measures should be assessed whenever possible as long term adherence to a weight loss diet is typically poor [77] and dietary recalls are prone to underreporting, although to a lesser extent than FFQs [78]. Additionally, the varied study durations, gender, age groups, protein types, and body composition assessments in this review are limitations, however, general conclusions can be drawn from the consistency in study findings per our theories.

## Conclusions

Most adults habitually consume 88 g or ~1.07 g/kg/day protein [6,79]. Per protein change theory, a 28.6% increase to a representative habitual protein intake would involve an increase of about 25–30 g/day or from 1.07 g/kg/day to 1.38 g/kg/day, which approximates the protein intake of most high protein groups in this review. Baseline protein intake should be known prior to deciding the level of protein intervention during a trial.

Designing studies with sufficient spread between group protein intakes would more likely assure a considerable difference between groups is achieved during the trial even with an expected degree of dietary non-compliance. Protein prescription proportional to bodyweight should become the norm in future studies versus% energy as should control for even distribution of protein across meals [75]. Finally, there is need for further examination of our theories in the context of change from higher baseline protein intakes.

Higher protein interventions were deemed successful when there was, on average, a 58.4% g/kg/day between group intake spread. In this review, the average change in habitual protein intake in weight loss studies showing higher protein to be more effective than control was +28.6%. These findings support our protein spread and change theories. Further research is needed to determine if there are specific spread and change thresholds.

## Abbreviations

RDA: Recommended dietary allowance; WHO: World Health Organization; FFQs: Food Frequency Questionnaire; GI: Glycemic index; g/kg/day: grams protein per kilogram per day.

## Competing interests

JDB and BMD are employees of USANA Health Sciences, Inc. USANA Health Sciences, Inc. had no role in the direction, data collection, analysis, interpretation, or writing of this review. USANA Health Sciences has provided for the article processing charge. The authors have no other competing interests to declare.

## Authors’ contributions

JDB designed the manuscript, collected and analyzed study data, wrote, and edited the manuscript. BMD provided manuscript direction and edited the manuscript. Both authors read and approved the final manuscript.

## Authors’ information

JDB holds an MS in Sports Dietetics, a BS in Exercise Science and is a Registered Dietitian and Senior Scientist for USANA Health Sciences, Inc. JDB is an Adjunct Professor to graduate students in the Division of Nutrition at the University of Utah. JDB has worked in the field with weight management clientele, collegiate, and professional athletes and in the lab researching shoulder biomechanics and the role of macronutrients in hypertension. Having reviewed protein metabolism literature, JDB’s current objective is to provide insight on scientific research based upon phenomena observed by practitioners in the field. BMD holds a PhD in Molecular and Cellular Biology from Oregon State University and has published numerous original scientific studies, most recently on the role of vitamin D in active populations. As Executive Director of Product & Technology Innovation, BMD oversees an expansive clinical studies program involving collaborations between USANA Health Sciences and several universities and private research institutions.

## Funding

JDB and BMD are employees of USANA Health Sciences, Inc. This review was prepared on company time.
